# A robust linear regression based algorithm for automated evaluation of peptide identifications from shotgun proteomics by use of reversed-phase liquid chromatography retention time

**DOI:** 10.1186/1471-2105-9-347

**Published:** 2008-08-19

**Authors:** Hua Xu, Lanhao Yang, Michael A Freitas

**Affiliations:** 1 Department of Molecular Virology Immunology and Medical Genetics, Comprehensive Cancer Center, the Ohio State University Medical Center, Columbus, 43210, OH, USA; 2 Department of Chemistry, the Ohio State University, Columbus, 43210, OH, USA

## Abstract

**Background:**

Rejection of false positive peptide matches in database searches of shotgun proteomic experimental data is highly desirable. Several methods have been developed to use the peptide retention time as to refine and improve peptide identifications from database search algorithms. This report describes the implementation of an automated approach to reduce false positives and validate peptide matches.

**Results:**

A robust linear regression based algorithm was developed to automate the evaluation of peptide identifications obtained from shotgun proteomic experiments. The algorithm scores peptides based on their predicted and observed reversed-phase liquid chromatography retention times. The robust algorithm does not require internal or external peptide standards to train or calibrate the linear regression model used for peptide retention time prediction. The algorithm is generic and can be incorporated into any database search program to perform automated evaluation of the candidate peptide matches based on their retention times. It provides a statistical score for each peptide match based on its retention time.

**Conclusion:**

Analysis of peptide matches where the retention time score was included resulted in a significant reduction of false positive matches with little effect on the number of true positives. Overall higher sensitivities and specificities were achieved for database searches carried out with MassMatrix, Mascot and X!Tandem after implementation of the retention time based score algorithm.

## Background

The science of proteomics encapsulates the large-scale identification, characterization and quantitation of proteins from biological samples. Mass spectrometry (MS) has been recognized as a powerful technique to study proteins. High-performance liquid chromatography (HPLC) coupled with tandem mass spectrometry (LC-MS/MS) is most commonly used in shotgun proteomics to resolve and identify proteolytic peptides generated from complex protein mixtures [1]. Peptide and protein identifications are usually derived from information contained in the tandem MS data. Automated database searching and *de novo *sequencing algorithms are routinely used to convert the MS/MS data into peptide and protein identifications [2]. Database search algorithms are more commonly used at this time due to their relatively low computational expense and higher compatibility with low mass accuracy and low quality MS/MS data [3,4].

It has also been recognized that the LC retention times of peptides are related to their sequences and can be used as complementary information for their identification and characterization [5]. Several methods have been developed to predict peptide retention times in reversed-phase liquid chromatography (RPLC) based on amino acid compositions and/or sequences [6-22]. High correlation between observed and predicted retention times for peptides in RPLC under different conditions has been achieved by use of these methods. Furthermore, these approaches can be combined with mass spectrometry to achieve better confidence in peptide identification than MS alone. For example, accurate mass tags combined with peptide retention time prediction has been effectively used by several groups to improve proteome characterization [23-26].

Peptide retention time prediction can also be used to refine and improve peptide identifications resulting from analysis of LC-MS/MS by database search software. In this way, false peptide matches from database search results can be minimized and true peptide matches can be confirmed with higher confidence. Krokhin *et al *reported an algorithm to refine the results obtained from the Global Proteome Machine[27] database searches [28]. In their approach, either internal or external standard peptides were used to estimate the regression parameters for the linear retention time prediction model used to refine their results. Strittmatter *et al *reported a post-database search method that evaluated peptide matches from SEQUEST[29] based upon their retention times. They reported a peptide retention time prediction model based on artificial neutral networks [15]. The prediction model was calibrated with highly reliable peptide matches from SEQUEST for each LC-MS/MS analysis [17]. An empirical discriminant score based on retention time and SEQUEST scores was also developed for peptide matches. It was shown that the number of reliable peptide matches was increased by use of peptide retention time information [17]. Klammer *et al *also developed an algorithm based on support vector regression to improve peptide identifications in tandem mass spectrometry by use of retention time prediction. As much as 50% of false peptide identifications in database search results from SEQUEST can be filtered and only 3% of true peptide matches were lost. The algorithm also trains the linear regression model dynamically for each data set [30].

We recently developed a robust linear regression based algorithm for automated evaluation of peptide identifications from database search programs based on retention time in RPLC. The algorithm extends the retention time prediction algorithm and its use for peptide identification in off-line LC-MS/MS by Krokhin *et al *[16,28]. The algorithm described here works for on-line LC-MS/MS experiments and eliminates the need of retention time prediction model calibration by use of internal or external standard peptides. The algorithm is generic and can be used to evaluate peptide matches from any database search program. It has been included in a database search program, MassMatrix [31], to perform automated data analysis. A post-hoc retention time analysis program LR_RT was also developed to analyze search results from other publicly available programs, such as Mascot and X!Tandem. Furthermore, a score algorithm was developed to provide a statistical score for each peptide match based on its predicted and observed retention times.

## Methods

### Sample preparation and mass spectrometry

Bovine histones were isolated from bovine thymus tissue as described by Sures *et al *[32,33]. The bovine histone mixture was digested by use of trypsin in 100 mM ammonium bicarbonate buffer (pH = 8.0). Enzymes were used in 25:1 ratio (substrate:enzyme) and the mixture was incubated at 37°C for two hours. The digested peptides were identified by use of data-dependent nano-LC-MS/MS on an LCQ Deca XP ion trap mass spectrometer (ThermoFisher, San Jose, CA, USA) as reported previously by Su *et al *[34]. In brief, 2.0 μL of bovine histone peptides at a total concentration of 0.1 μg/μL was injected and eluted off the capillary HPLC column (5 cm × 75 μm Pico Frit C18 column, 300 Å pore size, New Objective, Woburn MA) into the LCQ mass spectrometer at a flow rate of ~250 nL/min. Mobile phases A and B were water with 0.1% acetic acid and acetonitrile with 0.1% acetic acid respectively. A linear gradient of 5–50% of mobile phase B over 35 minutes was used. The total run time was 70 minutes.

### Database Search and Search Parameters

The .RAW data files obtained from the mass spectrometer were converted to mzXML files by use of ReAdW . Tandem mass spectra that were not derived from singly charged precursor ions were considered as both doubly and triply charged precursors. The mzXML file was searched by use of MassMatrix  against a database that contained both the bovine histone database and a reversed NCBInr human protein database as a decoy database. The search options were set as follows: i) No variable or fixed modifications; ii) Enzyme: trypsin; iii) Missed Cleavages = 3; iv) Peptide Length = 6 to 30 amino acid residues; and v) Mass tolerances of 2.0 Da and 0.8 Da for the precursor and product ions respectively. The data set was also evaluated by Mascot [35] and X!Tandem [27]. The search parameters were identical to those in MassMatrix. The search results from Mascot and X!Tandem were then analyzed by the post-hoc retention time analysis program, LR_RT  to obtain retention time based scores for each peptide match from the two search programs.

## Results and discussion

### Algorithm development

#### Overview

**Figure 1:**
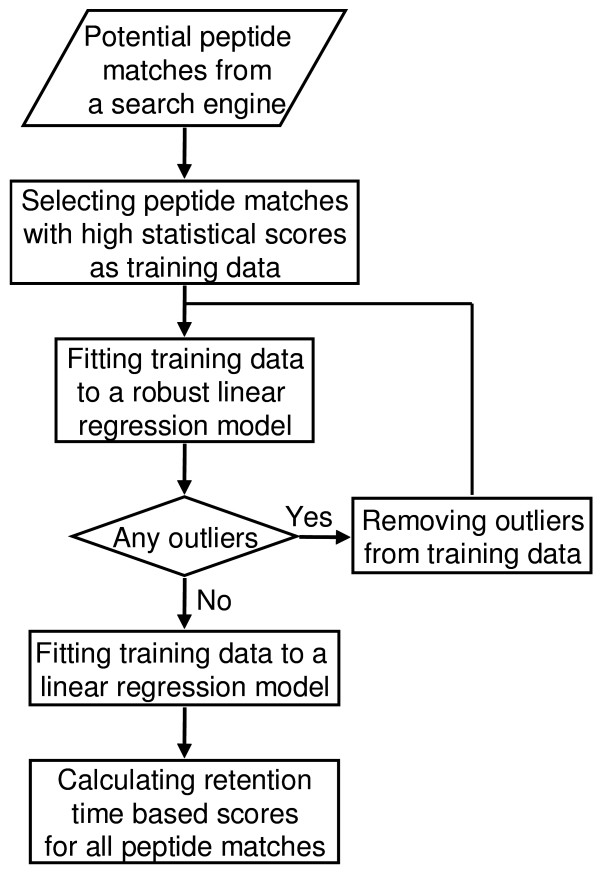
Flow diagram of the robust LR based algorithm for automated evaluation of peptide matches from a database search engine by their retention times.

Figure 1 shows the flow diagram for an algorithm that evaluates peptide matches based on their observed and predicted reversed-phase liquid chromatography retention times. The goal is to improve the confidence in peptide identification and lower the number of false positive matches returned by database search programs. In brief, the algorithm first creates a training data set from the peptide matches with the high statistical scores. These training data are then fitted to a robust linear regression (robust LR) model. Outliers due to false peptide matches in the training data set are removed by use of a recursive outlier-removal algorithm. The training data with outliers removed are then fitted to a linear regression (LR) model. A score for each peptide matches is then calculated.

A key advantage of this algorithm is that the LR model can be trained independently for each search. Thus there is no need to train or calibrate the LR model with internal or external standards for a given batch of samples. Furthermore, the algorithm is generic and can be used to evaluate peptide matches from any database search program. The algorithm can use different linear regression models for predicting peptide retention times under a variety of chromatographic conditions. For analysis of shotgun proteomic data sets, the linear regression model for peptide retention time prediction developed by Krokhin *et al *[16] was used. The model can be used to accurately predict retention times of tryptic peptides on reversed-phase (300 Å pore size) HPLC columns of various sizes with linear water-acetonitrile gradients containing trifluoroacetic acid, acetic acid, or formic acid as the ion-pairing agent [16]. The detailed implementation and performance of the algorithm are described in the next sections.

#### Linear regression model for predicting peptide retention times in RPLC

Retention times for true peptide matches identified by database search programs were assumed to follow a linear regression (LR) model: [16]

(1) *T *= *aH *+ *b *+ *ε*;

where *T *is the retention time of the peptide match, *H *is the calculated hydrophobicity of the peptide match, *a *and *b *are parameters for the linear model that depend on the RPLC column and elution gradient, and *ε *is the residual of the model. The hydrophobicity for a peptide is calculated from the peptide sequence by use of the model developed by Krokhin *et al *[16]. Their model was derived from the work of Guo *et al *[10,11] in which the hydrophobicity of a peptide is calculated by [16]

(2) $$H=\{\begin{array}{ll} K_{\text{L}}\left(\sum_{i=1}^{N}R_{C}^{i}+0.42R_{\text{cNt}}^{1}+0.22R_{\text{cNt}}^{2}+0.05R_{\text{cNt}}^{3}\right) & \text{if }H<38\\ 0.7K_{\text{L}}\left(\sum_{i=1}^{N}R_{C}^{i}+0.42R_{\text{cNt}}^{1}+0.22R_{\text{cNt}}^{2}+0.05R_{\text{cNt}}^{3}\right)+11.4 & \text{if }H\geq 38 \end{array}$$

where *K*_L _is the length correction coefficient of the peptide, *N *is the length of the peptide in terms of the number of amino acid residues, $\sum_{i=1}^{N}R_{C}^{i}$ is the sum of *R*_C _values for all amino acid residues of the peptide, and *R*^1 ^_cNt_, *R*^2 ^_cNt_, and *R*^3 ^_cNt _are the *R*_cNt _values for the first, second, and third amino acid residues from the N-terminus of the peptide respectively. The length correction coefficient *K*_L _is calculated by **eqn. 3**.

(3) $$K_{\text{L}}=\{\begin{array}{ll} 1-0.027\times (10-N) & \text{if }N<10\\ 1-0.014\times (N-20) & \text{if }N>20\\ 1 & \text{otherwise} \end{array}$$

The *R*_C _and *R*_cNt _values for the 20 common amino acid residues were reported previously by Krokhin *et al *[16].

#### Selection of training data

The retention time of the peptide match for each tandem mass spectrum was obtained from the mzXML or mzData file. We assume that retention times for true peptide matches will follow the LR model described in **eqn. 1**. The parameters of the LR model for true peptide matches are estimated from a training data set, which are then used to evaluate all peptide matches in the search result. In order to eliminate the need for LR model training by internal or external protein or peptide standards, the algorithm creates a training data set directly from the current database search results. This training data contains a selected number of peptide matches from the database search program with scores above a specified threshold. The accuracy and reliability of the LR model directly rely on the quality of this training data set. There are two major factors that affect model training: 1) size of the training data set and 2) false peptide matches included in the training data set that do not follow the linear regression model of the true matches. These false peptide matches should appear as outliers in our LR model and are referred to as outlier peptide matches. These outlier peptide matches have a negative impact on the calculation of parameters in the LR model. Increasing the threshold for statistical scores contained in the training data can minimize outlier peptide matches but will also significantly reduce the size of the training data set. By setting up moderate thresholds, a typical database search can generate training data sets containing 100 to 500 peptide matches. One challenge of this approach is that outlier peptide matches may be retained within the training data, especially for searches with large databases obtained at low mass accuracy.

#### Recursive outlier-removal algorithm

The LR model is vulnerable to outliers which distract the LR model training and lead to inaccurate results. As a result, it is crucial to remove as many outlier peptide matches as possible before model training. An algorithm based on robust LR was used to remove those outliers before model training. The algorithm required an iterative solution that is summarized as follows:

1. For each iteration step, a robust LR model is fitted to the training data set and gives robust estimates, $\tilde{\beta }$, of the parameters for the LR model in **eqn. 1 **(see **Appendix 2 **for details).

2. Calculate the residuals **e **= [*e*_1_, *e*_2_,⋯, *e*_n_]^T^_*n*×1 _based on the robust parameter estimates $\tilde{\beta }$ by **e **= **T **- **H **$\tilde{\beta }$.

3. Remove those data as outliers that have residuals outside the 95% confidence interval, i.e. *e*_*i *_≤ ${\tilde{\mu }}_{e}$ - 1.96 ${\tilde{\sigma }}_{e}$ or *e*_*i *_≥ ${\tilde{\mu }}_{e}$ + 1.96 ${\tilde{\sigma }}_{e}$, where ${\tilde{\mu }}_{e}$ is the median of the residuals, and ${\tilde{\sigma }}_{e}$ is equal to the median absolute of the residuals divided by 0.6745.

4. Repeat steps 1, 2 and 3 until no outliers are detected from the training data set in step 3 (Figure 1).

#### Score algorithm based on peptide retention time

After outliers are removed from the training data set, the LR model is fitted to the training data set by ordinary least squares to give estimates of the parameters (**eqn. A.1**). The predicted retention time $\hat{T}$ for a peptide with the calculated hydrophobicity *H *is given by **eqn. A.2**. The prediction error, *D*, and the absolute error, Δ, of the predicted retention time for the peptide are respectively defined as

(4) $$D=T-\hat{T}.$$

and

(5) $$\Delta =\left|D\right|=\left|T-\hat{T}\right|.$$

The observed error of the prediction, *δ*, is calculated by

(6) $$\delta =\left|T_{\text{obs}}-\hat{T}\right|.$$

where *T*_obs _is the observed retention time of the peptide. The score based on peptide retention time, *C*_RT_, is defined as the probability that the theoretical absolute error Δ for a peptide match is greater than or equal to the observed absolute error *δ*, given that the peptide is a true match, i.e.

(7) $$\begin{array}{c} C_{\text{RT}}=P(\Delta \geq \delta |\text{peptide match is true})\\ =P(D\geq \delta |\text{peptide match is true})+P(D\leq -\delta |\text{peptide match is true}) \end{array}$$

Given the two assumptions that all true peptide matches follow the linear regression model in **eqn. 1 **and the linear regression model is a normal error regression model, $\frac{D}{{\text{SE}}_{\hat{T}}}$ follows a t distribution with degrees of freedom of *n *- 2 for true peptides (see **Appendix 1 **for details). The score is calculated by the following equation.

(8) $$\begin{array}{c} C_{\text{RT}}=P(D\geq \delta |\text{peptide match is true})+P(D\leq -\delta |\text{peptide match is true})\\ =P(\frac{D}{SE_{\hat{T}}}\geq \frac{\delta }{SE_{\hat{T}}}|\text{peptide match is true})+P(\frac{D}{SE_{\hat{T}}}\leq -\frac{\delta }{SE_{\hat{T}}}|\text{peptide match is true})\\ =2\times \left[1-F_{\text{t}(n-2)}\left(\frac{\delta }{SE_{\hat{T}}}\right)\right] \end{array}$$

where ${\text{SE}}_{\hat{T}}$ is the standard error of the predicted retention time given in **eqn. A.9**, *F*_t(*n*-2)_(*x*) is the cumulative density function of the t distribution with degrees of freedom of *n *- 2. Smaller *δ *gives higher *C*_RT _score and indicates a higher confidence for the peptide match.

All cumulative density functions of any continuous distributions, including *F*_t(*n*-2)_(*x*) for -∞ <*x *< ∞, follows a continues uniform distribution over the interval of [0, 1]. The distribution of $t=F_{\text{t}(n-2)}\left(\frac{\delta }{SE_{\hat{T}}}\right)=1-\frac{C_{\text{RT}}}{\text{2}}$ is a continuous uniform distribution over the interval of [0.5, 1] due to *δ *> 0. Its probability density function is given in **eqn. 9**.

(9) $$f_{t}(t)=\{\begin{array}{ll} 2 & 0.5\leq t\leq 1.0\\ 0 & \text{otherwise} \end{array}$$

The probability density function of *C*_RT _for true peptide matches can be derived from that of $t=F_{\text{t}(n-2)}\left(\frac{\delta }{SE_{\hat{T}}}\right)=1-\frac{C_{\text{RT}}}{\text{2}}$

(10) $$f_{C_{\text{RT}}}(C_{\text{RT}})=\left|\frac{dt}{dC_{\text{RT}}}\right|f_{t}(t)=\left|\frac{d}{dC_{\text{RT}}}\left(1-\frac{C_{\text{RT}}}{\text{2}}\right)\right|f_{t}\left(1-\frac{C_{\text{RT}}}{\text{2}}\right)=\frac{1}{2}f_{t}\left(1-\frac{C_{\text{RT}}}{\text{2}}\right)$$

Substitution of **eqn. 9 **into **eqn. 10 **yields the following.

(11) $$f_{C_{\text{RT}}}(C_{\text{RT}})=\{\begin{array}{ll} 1 & 0\leq C_{\text{RT}}\leq 1\\ 0 & \text{otherwise} \end{array}$$

Thus, *C*_RT _follows a continuous uniform distribution over the interval [0, 1]. Consequently, its cumulative density function is given in **eqn. 12**.

(12) $$F_{C_{\text{RT}}}(C_{\text{RT}})=\{\begin{array}{ll} 0 & C_{\text{RT}}<0\\ C_{\text{RT}} & 0\leq C_{\text{RT}}\leq 1\\ 1 & C_{\text{RT}}>1 \end{array}$$

The theoretical distribution of *C*_RT _for random peptide matches is unknown and varies from one search to another.

### Automate evaluation of peptide matches based on their retention times

The retention time score algorithm is included as part of the MassMatrix database search program to perform automated evaluation of peptide matches. Due to the robustness of the algorithm, the score threshold for selection of training data does not significantly affect the model training and results. The score threshold for selection of training data for the algorithm in MassMatrix was set to be ≥ 8.0 for both pp and pp2 scores [31] and ≥ 2.0 for pp_tag _score [36]. MassMatrix takes the mzXML, mzData and MGF data files as input data formats. The retention time based algorithm automatically scores peptide matches if the input data file is either mzXML or mzData. The retention time based algorithm is not used if the input data file is a MGF file due to the fact that MGF files lack retention times.

### Post-hoc retention time scoring of other database search programs

The retention time based algorithm described herein is generic and a post-hoc retention time analysis program for all other database search programs, LR_RT, was developed to perform automated post-search evaluation of the peptide matches. The post-hoc analysis requires the original mzData or mzXML file along with a tab or space delimited .txt file of the search results. The search result file must contain the scan number, peptide sequence and score information for each peptide match. The program was tested on Mascot and X!Tandem search results. Search results in the tab delimited .txt files can be obtained from Mascot html search results or X!Tandem pepXML search results by use of Perl scripts available at . Score thresholds for selection of training data for the retention time based algorithm are set to be ≥ 30 and ≤ 0.1 for Mascot and X!Tandem results respectively.

### Evaluation of the robust linear regression based algorithm

#### MassMatrix automated evaluation of peptide matches based on their retention times

The robust LR based algorithm built in MassMatrix was evaluated against experimental LC-MS/MS data from bovine histone digests acquired by use of a LCQ Deca XP+ MS. The data set contained 3166 tandem mass spectra and was searched against a database that contained a bovine histone database and the NCBInr reversed human protein database as decoy sequences. The complete list of peptide matches is provided in the additional file [see Additional file 1]. The decoy database was much larger than the bovine histone database and created ~1000 times as many theoretical peptides as the bovine histone database. False positive peptide matches from the bovine histone database were thus assumed to be negligible [37,38]. As a result peptide matches returned from the bovine histone database were considered as true positives (TPs) while those from the decoy database were considered to be false positives (FPs).

**Figure 2:**
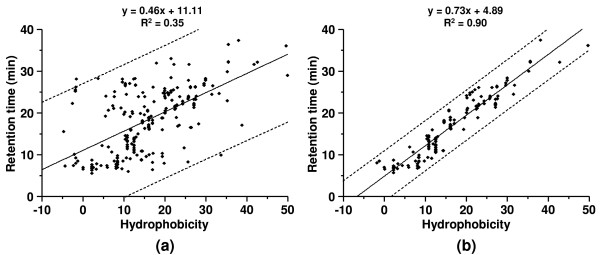
Scatter plots of the training data set (a) before and (b) after removal of outliers by the recursive outlier-removal algorithm for the bovine histone data set. The fitted LR equation along with their coefficients of determination (R^2 ^values) is shown at the top of the figures. The solid and dashed lines represent the fitted LR models and their 99% confidence bands respectively.

Figure 2a shows the scatter plot of the original 254 peptide matches selected as training data. From the figure it is obvious that outlier peptide matches are present in the training data. The original training data set was fitted to the LR model without removal of any outliers. The coefficient of determination (R^2 ^value) for the LR model was 0.35, which indicated a poor correlation between peptide retention time and peptide hydrophobicity. Furthermore, the 99% confidence band from the linear model is too wide to be useful for scoring peptide matches. As expected, the training data set chosen by the database search program cannot be directly used to train the LR model due to outlier positive matches included in the training data.

Figure 2b shows a scatter plot of the training data set that contained 143 peptide matches after removal of outliers by the recursive outlier-removal algorithm described above. Outlier removal resulted in a strong linear relationship between the retention times and the hydrophobicities of peptide matches in the training data. The training data set was fitted to the LR model, and the R^2 ^value was improved from 0.35 to 0.90 after removal of the outliers. Furthermore, the 99% confidence band of the LR models was much narrower after the outlier removal by the algorithm.

**Figure 3:**
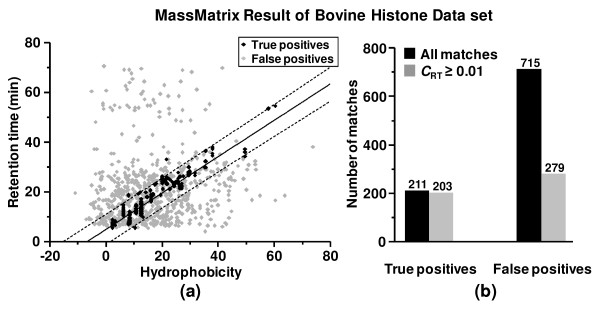
(a) Scatter plot of all true and false peptide matches from the search in MassMatrix for the bovine histone data set. The solid line represents the LR model trained by the training data set after outlier removal (see **Figure 2b**). The dashed lines represent the 99% confidence band of the model. (b) Histogram of number of all true and false positive peptide matches and those lying within the 99% confidence band of the linear model, i.e. with *C*_RT _≥ 0.01.

**Figure 4:**
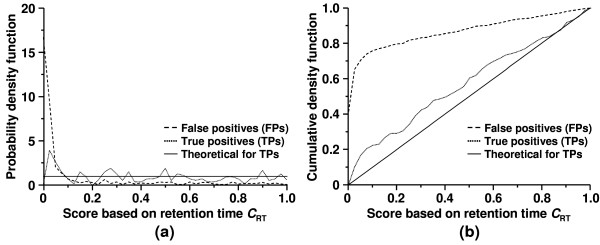
Distributions of retention time based score *C*_RT _for true and false positive peptide matches of the bovine histone data set: (a) probability density function $f_{C_{\text{RT}}}(C_{\text{RT}})$ and (b) cumulative density function $F_{C_{\text{RT}}}(C_{\text{RT}})$. Theoretical distributions of true positive matches are indicated by the solid lines.

The accuracy and robustness of the algorithm is illustrated in the scatter plot for all peptide matches from the search (Figure 3a). The solid and dashed lines in the figure represent the LR model and its 99% confidence band fitted to the training data set with a size of 143 after outlier removal. The key concern is that the application of the LR model reduces false positives but not at the expense of true positives. In fact 203 of 211 (96.21%) true peptide matches were observed within the 99% confidence band, i.e. with *C*_RT _≥ 0.01 (Figure 3b). In contrast, 279 of 715 (39.02%) false peptide matches were found within the 99% confidence band. Therefore, the majority (60.98%) of false peptide matches were filtered by the application of the LR model described herein while only 3.79% of true positive peptide matches were lost. The distributions of retention time based scores, *C*_RT_, for true positive and false peptide matches are shown in Figure 4. The score distribution for true peptide matches was close to that for the expected theoretical distribution described by **eqn. 11 & 12**. False peptide matches had much lower scores than true peptide matches where majority of the false matches had scores less than 0.01.

The algorithm was also evaluated by use of two publicly available LC-MS/MS data sets from significantly more complex samples. The first data set was created by use of LC-MS/MS on an LCQ ion trap mass spectrometer from a tryptic digest of a proteome sample from Deinococcus radiodurans MR-1 gram-positive bacteria. The data set (Dataset_021014.RAW for Deinococcus radiodurans data) along with the experimental details can be obtained at . The data set was searched by use of the MassMatrix database search program against a database that contained both the Deinococcus radiodurans database and a dominant reversed NCBInr human protein database used as a decoy database. The second data set was created by use of 2D-LC-MS/MS on an LCQ Deca XP+ mass spectrometer from the tryptic digest of a human proteome sample. The sample was separated by a SCX column in 11 salt steps and the fraction from each step was analyzed by a C18 RPLC-MS/MS. Eleven MS/MS data sets were generated. The data set that was created from the first fraction that contained the greatest number of MS/MS scans among all 11 data sets was used in our evaluation. The data set and the experimental details can be found at [39]. The data set was searched against a database with a target NCBInr human database and a dominant decoy database. The dominant decoy database contained ten randomized NCBInr human database and one reversed human database. The search parameters for both data sets were the same as those used for the bovine histone data set.

**Figure 5:**
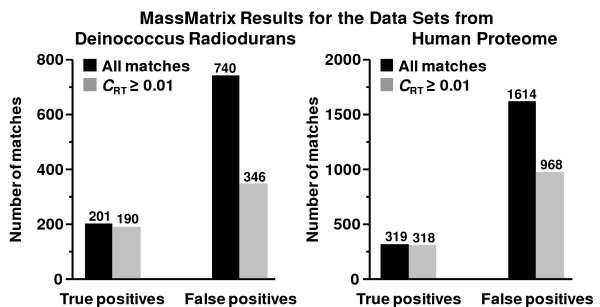
Histogram of number of all true and false positives peptide matches as well as those lying within the 99% confidence band of the linear model, i.e. with *C*_RT _≥ 0.01, for the Deinococcus radiodurans and human proteome data sets.

The algorithm independently trained the peptide retention time prediction LR model for both the Deinococcus radiodurans data set and the human proteome data set. The R^2 ^of the linear regression models for the two data sets were 0.90 and 0.93. As shown in Figure 5a, 53.24% of FPs was filtered at a threshold of 0.01 for *C*_RT _with a loss of 5.47% of TPs for the Deinococcus radiodurans data set. For the human proteome data set, 40.02% of FPs was filtered as loss of 0.31% TPs. The results suggest that the algorithm implemented in MassMatrix can be effectively used to reduce false positives of database search results for LC-MS/MS proteomic data from complex samples.

#### Test of the assumptions of the algorithm

There are two assumption involved in the algorithm. The first is that all true positives follow the linear regression model. It can be seen from the previous discussion that this assumption was violated. However, this departure from the first assumption was small and only caused small losses (0.31 to 5.47%) of TPs.

**Figure 6:**
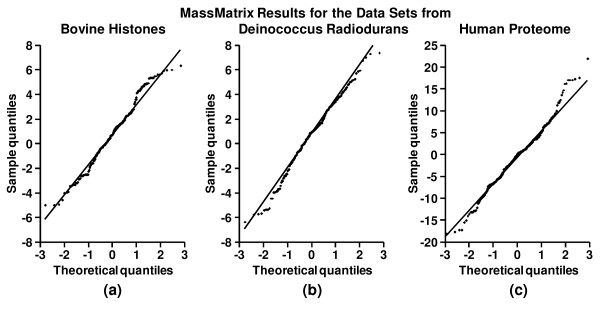
The Q-Q plots of all the residuals of the true positives following the linear regression model from the MassMatrix search results for the bovine histone data set, the Deinococcus radiodurans data set, and the human proteome data set.

The second assumption involved in the algorithm is that the linear regression model in **eqn. 1 **is a normal error model, i.e., the residuals of the TPs that follow the linear regression model are normally distributed. This assumption was tested by the quantile-quantile plots (Q-Q plots) of all the residuals of the TPs following the linear regression model, i.e. with *C*_RT _≥ 0.01 for the three data sets. The three Q-Q plots for the three data sets are shown in Figure 6. It can be seen that the Q-Q plots for the bovine histone and Deinococcus radiodurans data sets formed a linear pattern and the normality assumption was valid (Figures 6a &6b). For the human proteome data set, there was a slight departure from the normality as shown in Figure 6c. However, the small departure from the normality is not significant and does not create any serious concerns. Overall these results demonstrate that there was no significant departure from normality of the residuals for TPs that follow the linear model.

#### Post-hoc retention time analysis program for other database search programs

**Figure 7:**
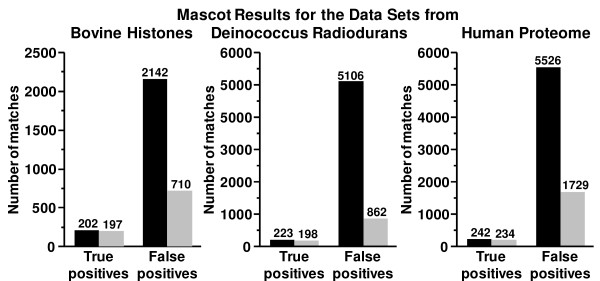
Histograms of number of all true and false positive peptide matches as well as those with *C*_RT _≥ 0.01 of the Mascot search results for the bovine histone data set, the Deinococcus radiodurans data set, and the human proteome data set.

**Figure 8:**
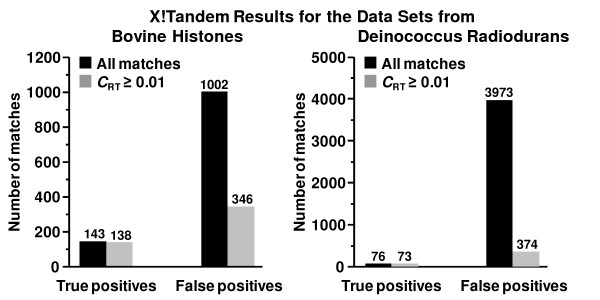
Histograms of number of all true and false positive peptide matches as well as those with *C*_RT _≥ 0.01 of the X!Tandem search results for the bovine histone and Deinococcus radiodurans data sets.

The post-hoc retention time analysis program, LR_RT, was used to evaluate Mascot and X!Tandem search results for the bovine histone data set with the same database and search parameters as used with the MassMatrix search. Peptide matches from these two database search programs were evaluated by our retention time based algorithm, LR_RT. A retention time based score, *C*_RT_, was calculated for each peptide match. The complete lists of peptide matches along with their *C*_RT _scores are provided in the additional files [see Additional files 2 &3]. The algorithm independently trained the peptide retention time prediction LR model for both the Mascot and X!Tandem search results. Similar to the case for MassMatrix, search results for Mascot and X!Tandem were significantly improved after retention time scoring. The final R^2 ^values from the LR models were 0.91 and 0.94 for Mascot and X!Tandem results respectively. More importantly, the LR_RT program was able to significantly reduce the number of false positives for both Mascot and X!Tandem search results. For Mascot research results, 66.85% of false positives were filtered with a threshold of 0.01 for *C*_RT_, whereas only 2.48% of true positives were filtered with the same threshold (Figure 7a). For X!Tandem results, 65.47% of false positives were filtered with a loss of 3.50% of true positives (Figure 8a). Therefore, the majority of the false peptide matches from the two programs can be filtered by the retention time based score algorithm with a modest negative impact on the number of true positives.

The post-hoc retention time analysis program was also evaluated by Mascot and X!Tandem search results from the Deinococcus radiodurans and human proteome data sets from complex samples. The databases and search parameters in Mascot and X!Tandem were the same as those used in the MassMatrix searches for the two data sets. For the Mascot searches of the Deinococcus radiodurans and human data sets (Figures 7b &7c) and the X!Tandem search of the Deinococcus radiodurans data set (Figure 8b), the algorithm also effectively reduced false positives with small losses of true positives. However, the algorithm was not applicable to the X!Tandem search of the human data set. This was due to the fact that X!Tandem did not return a significant number of true positives for the data set. The number of peptide matches with expectation value ≤ 0.1 from the target database of the search was 14, which was not enough for peptide retention time model training.

#### ROC analysis

**Figure 9:**
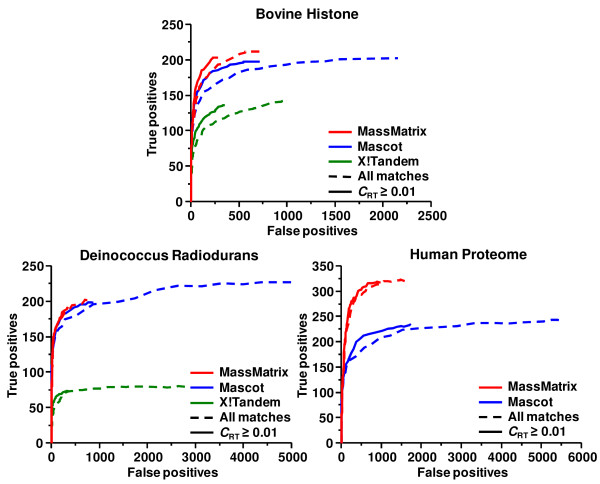
ROC curves of MassMatrix, Mascot and X!Tandem search results before (dashed line) and after (solid line) filtering peptide matches with *C*_RT _< 0.01 for the bovine histone data set, the Deinococcus radiodurans data set, and the human proteome data set.

Figure 9 displays the receiver operating characteristic (ROC) curves for the MassMatrix, Mascot and X!Tandem search results of the three data sets before and after removal of peptide matches with *C*_RT _< 0.01. For the human data set, the X!Tandem result was not shown due to the fact that X!Tandem did not return a significant number of true positives. It can be seen that higher sensitivities and specificities were achieved after filtering peptide matches with insignificant *C*_RT _scores for the searches of the bovine histone and Deinococcus radiodurans data sets in all three programs and the searches of the human proteome data set in MassMatrix and Mascot. Therefore, the false positive rates of search results in MassMatrix, Mascot and X!Tandem can be significantly lowered by including the new score algorithm based on peptide retention time.

## Conclusion

An algorithm based on robust LR has been developed for automated evaluation of peptide matches from database searches by use of peptide retention time in reversed-phase HPLC. The recursive outlier-removal algorithm based on robust LR enables the algorithm to train the LR model on the fly for each search thus the need for internal or external protein or peptide standards is eliminated. The LR model for peptide retention in RPLC developed by Krokhin *et al *[16] was adopted in the current implementation of the algorithm.

The algorithm was implemented in the MassMatrix database search program and evaluated with a LC-MS/MS data set of bovine histones obtained on a LCQ Deca XP mass spectrometer. The R^2 ^value for LR model was improved from 0.35 to 0.90 after outlier removal. The majority (96.21%) of true peptide matches fell within the 99% confidence band for the trained LR model, whereas only 39.02% of false peptide matches fell in the same 99% confidence band. By use of this approach the majority (60.98%) of the false peptide matches can be filtered from the results based on retention time while only losing 3.79% of the true positive peptide matches.

A post-hoc retention time analysis program, LR_RT, was also developed to analyze peptide matches from other database search programs. The program was tested on Mascot and X!Tandem search results for the bovine histone data set. More than 60% of false positives in Mascot and X!Tandem search results were filtered by the program with a loss of less than 3.5% for true positives.

The algorithm was also tested on two publicly available data sets from complex samples. For the data set from a Deinococcus radiodurans proteome sample, the algorithm was able to reduce the majority of false positives at a small loss of true positives for searches in MassMatrix, Mascot and X!Tandem. For the data set from a human proteome sample, the algorithm could still effectively reduce false rates for searches in MassMatrix and Mascot. For the search of that data set in X!Tandem, the algorithm was not applicable due to the fact that X!Tandem was not able to catch a significant number of true positives.

A statistical score algorithm was developed for ranking peptide matches based on predicted and observed retention times. The score distribution for true peptide matches was close to its theoretical distribution, which indicates that the LR model trained by the robust LR based algorithm represents the true linear relationship between the peptide retention times in RPLC and their calculated hydrophobicities. False peptide matches tend to have much lower scores than true matches, and the majority of the false matches have scores less than 0.01. This score enables differentiation between true and false matches based on retention time. After removal of peptide matches with insignificant scores based on retention time, higher sensitivities and specificities were achieved and the false positive rates of the searches were significantly lowered as shown by the ROC analysis for all the three database search programs.

## Availability and requirements

Project name: MassMatrix Retention Time Analysis.

Project home page: .

Operating systems: Windows, Linux.

Programming language: ANSI C++.

Other requirements: None.

License: None.

Any restrictions to use by non-academics: None.

## Appendix

### 1. Linear Regression

For the LR model described in **eqn. 1**, the ordinary least-square estimates of the parameters from a training data set of sample size *n *are given by

(A1) $$\hat{\beta }={\left(H^{\text{T}}H\right)}^{-1}H^{\text{T}}T;$$

where estimated parameter matrix $\hat{\beta }={\left[\begin{array}{c} \hat{a}\\ \hat{b} \end{array}\right]}_{2\times 1}$, and matrices for the training data set $H={\left[\begin{array}{cc} 1 & H_{1}\\ 1 & H_{2}\\ \vdots & \vdots \\ 1 & H_{n} \end{array}\right]}_{n\times 2}$, and $T={\left[\begin{array}{c} T_{1}\\ T_{2}\\ \vdots \\ T_{n} \end{array}\right]}_{n\times 1}$. *H*_i _and *T*_i _are the hydrophobicity and experimental retention time of the peptide match *i *in the training data set. The predicted retention time for a peptide with hydrophobicity *H*, is calculated by

(A2) $$\hat{T}=h\hat{\beta }=\hat{a}+\hat{b}H;$$

where **h **= [1, *H*]_1×2_.

A regression model in **eqn. 1 **that assumes the residual *ε *follows an independent N(0, *σ*^2^) is called a normal error regression model. For this type of model, the variance of *T *is

(A3) Var(*T*) = *σ*^2^

According to **eqn. A.1 **and **A.2**, the variance of $\hat{\beta }$ is

(A4) $$\begin{array}{c} Var(\hat{\beta })=Var\{{\left(H^{\text{T}}H\right)}^{-1}H^{\text{T}}T\}=\left\{{\left(H^{\text{T}}H\right)}^{-1}H^{\text{T}}\right\}Var(T){\left\{{\left(H^{\text{T}}H\right)}^{-1}H^{\text{T}}\right\}}^{T}\\ =\left\{{\left(H^{\text{T}}H\right)}^{-1}H^{\text{T}}\right\}\sigma^{2}I{\left\{{\left(H^{\text{T}}H\right)}^{-1}H^{\text{T}}\right\}}^{T}=\sigma^{2}{\left(H^{\text{T}}H\right)}^{-1} \end{array}$$

and the variance of $\hat{T}$ is

(A5) $$Var(\hat{T})=Var(h\hat{\beta })=hVar(\hat{\beta })h^{T}=\sigma^{2}h{\left(H^{\text{T}}H\right)}^{-1}h^{\text{T}}$$

For a peptide match that follows the linear model, the variance of the prediction error

(A6) $$Var(T-\hat{T})=Var(T)+Var(\hat{T})=\sigma^{2}+\sigma^{2}h{\left(H^{\text{T}}H\right)}^{-1}h^{\text{T}}=\sigma^{2}\times \left\{1+h{\left(H^{\text{T}}H\right)}^{-1}h^{\text{T}}\right\}$$

The estimate of *σ*^2^, MSE, is calculated by

(A7) $$\text{MSE}=\frac{1}{n-2}{\left(T-H\hat{\beta }\right)}^{\text{T}}\left(T-H\hat{\beta }\right)$$

Therefore, the variance of the prediction error, *T *- $\hat{T}$, can be estimated by

(A8) $$\hat{V}ar(T-\hat{T})=\text{MSE}\times \left\{1+h{\left(H^{\text{T}}H\right)}^{-1}h^{\text{T}}\right\}$$

and the standard error for the predicted retention time is estimated by

(A9) $${\text{SE}}_{\hat{T}}=\sqrt{\hat{V}ar(T-\hat{T})}=\sqrt{\text{MSE}\times \left\{1+h{\left(H^{\text{T}}H\right)}^{-1}h^{\text{T}}\right\}}$$

Due to the normality assumption of the residuals, the prediction error follows a normal distribution. Therefore, $\frac{T-\hat{T}}{{\text{SE}}_{\hat{T}}}$ is a t distribution with degrees of freedom of (*n *- 2).

### 2. Robust Linear Regression

Ordinary least-square estimates of LR models can be severely affected by outliers that may lead to incorrect results. Another approach called robust LR is less vulnerable to outliers. A commonly used solution of a robust LR model involves an iterative method which is summarized as follows:[40]

1. The ordinary least-square estimates of the LR model from **eqn. A.1 **are obtained as initial estimates of the regression parameters, ${\tilde{\beta }}_{0}$.

2. At the *i*^th ^iteration step, calculate residuals based on the parameter estimates from the previous *i*-1^th ^iteration, ${\tilde{\beta }}_{i-1}$,

(A10) $$e=T-H{\tilde{\beta }}_{i-1};$$

where, **e **= [*e*_1_, *e*_2_,⋯, *e*_*n*_]^T^_*n*×1_. Calculate associated weights by

(A11) $$w={\left[\begin{array}{c} w_{1}\\ w_{2}\\ \vdots \\ w_{n} \end{array}\right]}_{n\times 1}={\left[\begin{array}{c} w_{B}(e_{1})\\ w_{B}(e_{2})\\ \vdots \\ w_{B}(e_{n}) \end{array}\right]}_{n\times 1};$$

where w_B_(*x*) is Bisquare weight function,

(A12) $$w_{B}(x)=\{\begin{array}{cc} {\left[1-{\left(\frac{x}{k}\right)}^{2}\right]}^{2} & \text{for }\left|x\right|\leq k\\ 0 & \text{for }\left|x\right|>k \end{array};$$

and k is the tuning constant for the function that is given by *k *= 4.685 ${\tilde{\sigma }}_{e}$. ${\tilde{\sigma }}_{e}$ is the robust estimate of the standard deviation of the residuals, and it is equal to the median absolute of the residuals divided by 0.6745.

3. Calculate the weighted-least-squares estimates

(A13) $${\tilde{\beta }}_{i}={\left(H^{\text{T}}WH\right)}^{-1}H^{\text{T}}WT;$$

where, $W=diag(w)={\left[\begin{array}{cccc} w_{1} & 0 & 0 & 0\\ 0 & w_{2} & 0 & 0\\ \vdots & \vdots & \ddots & \vdots \\ 0 & 0 & 0 & w_{n} \end{array}\right]}_{n\times n}.$.

4. Repeat steps 2 and 3 until the parameter estimates converge.

The converged estimates, $\tilde{\beta }$, are the solution for the robust LR model.

## Authors' contributions

HX designed the algorithm and drafted the manuscript. MAF was the principle investigator and initiated the project. MAF also provided overall guidance of the project and revised the manuscript critically. LY prepared the peptide sample and collected the LC-MS/MS data. All authors have read and approved the final manuscript.

## Supplementary Material

Additional file 1 Peptide matches from MassMatrix search result for the bovine histone data set. The complete list of peptide matches along with their *C*_RT _scores from the search in MassMatrix for the bovine histone data set. Click here for file

Additional file 2 Peptide matches from Mascot search result for the bovine histone data set. The complete list of peptide matches along with their *C*_RT _scores from the search in Mascot for the bovine histone data set. Click here for file

Additional file 3 Peptide matches from X!Tandem search result for the bovine histone data set. The complete list of peptide matches along with their *C*_RT _scores from the search in X!Tandem for the bovine histone data set. Click here for file

## References

[B1] Aebersold R, Mann M (2003). Mass spectrometry-based proteomics. Nature.

[B2] Nesvizhskii AI, Aebersold R (2004). Analysis, statistical validation and dissemination of large-scale proteomics datasets generated by tandem MS. Drug Discov Today.

[B3] Sadygov RG, Cociorva DC, Yates JR (2004). Large-scale database searching using tandem mass spectra: Looking up the answer in the back of the book. Nature Methods.

[B4] Kapp EA, Schütz F, Connolly LM, Chakel JA, Meza JE, Miller CA, Fenyo D, Eng JK, Adkins JN, Omenn GS, Simpson RJ (2005). An evaluation, comparison, and accurate benchmarking of several publicly available MS/MS search algorithms: sensitivity and specificity analysis. Proteomics.

[B5] Shinoda K, Sugimoto M, Tomita M, Ishihama Y (2008). Informatics for peptide retention properties in proteomics LC-MS. Proteomics.

[B6] Meek JL (1980). Prediction of peptide retention times in high-pressure liquid chromatography on the basis of amino acid composition. Proc Natl Acad Sci USA.

[B7] Meek JL, Rossetti ZL (1981). Factors affecting retention and resolution of peptides in high-performance liquid-chromatography. J Chromatogr.

[B8] Browne CA, Bennett HPJ, Solomon S (1982). The isolation of peptides by high-performance liquid-chromatography using predicted elution positions. Anal Biochem.

[B9] Sasagawa T, Okuyama T, Teller DC (1982). Prediction of peptide retention times in reversed-phase high-performance liquid-chromatography during linear gradient elution. J Chromatogr.

[B10] Guo D, Mant CT, Taneja AK, Hodges RS (1986). Prediction of peptide retention times in reversed-phase high-performance liquid chromatography II. Correlation of observed and predicted peptide retention times factors and influencing the retention times of peptides. J Chromatogr A.

[B11] Guo D, Mant CT, Taneja AK, Parker JMR, Hodges RS (1986). Prediction of peptide retention times in reversed-phase high-performance liquid chromatography I. Determination of retention coefficients of amino acid residues of model synthetic peptides. J Chromatogr A.

[B12] Mant CT, Burke TWL, Black JA, Hodges RS (1988). Effect of peptide-chain length on peptide retention behavior in reversed-phase chromatography. J Chromatogr.

[B13] Sakamoto Y, Kawakami N, Sasagawa T (1988). Prediction of peptide retention times. J Chromatogr.

[B14] Palmblad M, Ramstrom M, Markides KE, Hakansson P, Bergquist J (2002). Prediction of chromatographic retention and protein identification in liquid chromatography/mass spectrometry. Anal Chem.

[B15] Petritis K, Kangas LJ, Ferguson PL, Anderson GA, Pasa-Tolic L, Lipton MS, Auberry KJ, Strittmatter EF, Shen Y, Zhao R, Smith RD (2003). Use of artificial neutral networks for the accurate prediction of peptide liquid chromatography elution times in proteome analysis. Anal Chem.

[B16] Krokhin OV, Craig R, Spicer V, Ens W, Standing KG, Beavis RC, Wilkins JA (2004). An improved model for prediction of retention times of tryptic peptides in ion pair reversed-phase HPLC. Mol Cell Proteomics.

[B17] Strittmatter EF, Kangas LJ, Petritis K, Mottaz HM, Anderson GA, Shen Y, Jacobs JM, Camp II DG, Smith RD (2004). Application of peptide LC retention time information in a discriminant function for peptide identification by tandem mass spectrometry. J Proteome Res.

[B18] Baczek T, Wiczling P, Marszall M, Heyden YV, Kallszan R (2005). Prediction of peptide retention at different HPLC conditions from multiple linear regression models. J Proteome Res.

[B19] Wang Y, Gu X, Zhang J, Zhang XM (2005). Prediction of peptid retention in RPLC. Chromatographia.

[B20] Gorshkov AV, Tarasova IA, Evreinov VV, Savitski MM, Nielsen ML, Zubarev RA, Gorshkov MV (2006). Liquid chromatography at critical conditions: Comprehensive approach to sequence-dependent retention time prediction. Anal Chem.

[B21] Petritis K, Kangas LJ, Yan B, Monroe ME, Strittmatter EF, Qian W, Adkins JN, Moore RJ, Xu Y, Lipton MS, Camp II DG, Smith RD (2006). Improved peptide elution time prediction for reversed-phase liquid chromatography-ms by incorporating peptide sequence information. Anal Chem.

[B22] Tripet B, Cepeniene DC, Kovacs JM, Mant CT, Krokhin OV, Hodges RS (2007). Requirements for prediction of peptide retention time in reversed-phase high-performance liquid chromatography: Hydrophilicity/hydrophobicity of side-chains at the N- and C-termini of peptides are dramatically affected by the end-groups and location. J Chromatogr A.

[B23] May D, Fitzgibbon M, Liu Y, Holzman T, Eng J, Kemp CJ, Whiteaker J, Paulovich A, McIntosh M (2007). A platform for accurate mass and time analysis of mass spectrometry data. J Proteome Res.

[B24] Norbeck AD, Monroe ME, Adkins JN, Anderson KK, Daly DS, Smith RD (2005). The utility of accurate mass and LC elution time information in the analysis of complex proteomes. J Am Soc Mass Spectrum.

[B25] Jaitly N, Monroe ME, Paetyuk VA, Clauses TRW, Adkins JN, Smith RD (2006). Robust algorithm for alignment of liquid chromatography-mass spectrometry analyses in an accurate mass and time tag data analysis pipeline. Anal Chem.

[B26] Palmblad M, Ramstrom M, Bailey CG, McCutchen-Maloney SL, Bergquist J, Zeller LC (2004). Protein identification by liquid chromatography-mass spectrometry using retention tiem prediction. J Chromatogr B Analyt Technol Biomed Life Sci.

[B27] Craig R, Cortens JP, Beavis RC (2004). Open source system for analyzing, validating, and storing protein identification data. J Proteome Res.

[B28] Krokhin OV, Ying S, Cortens JP, Ghosh D, Spicer V, Ens W, Standing KG, Beavis RC, Wilkins JA (2006). Use of peptide retention prediction for protein identification by off-line reversed-phase HPLC-MALDI MS/MS. Anal Chem.

[B29] Eng JK, McCormack AL, Yates JR (1994). An approach to correlate tandem mass spectral data of peptides with amino acid sequences in a protein database. J Am Soc Mass Spectrom.

[B30] Klammer AA, Yi X, MacCoss MJ, Noble WS (2007). Improving tandem mass spectrum identification using peptide retention tiem prediction across diverse chromatography conditions. Anal Chem.

[B31] Xu H, Freitas AF (2007). A high mass accuracy sensitive probability based scoring algorithm for database searching of tandem mass spectrometry data. BMC Bioinformatics.

[B32] Sures I, Gallwitz D (1980). Histone-specific acetyltransferases from calf thymus. isolation, properties, and substrate specificity of three different enzymes. Biochem.

[B33] Zhang LW, Freitas MA, Wickham J, Parthun MR, Klisovic MI, Marcucci G, Byrd JC (2004). Differential expression of histone post-translational modifications in acute myeloid and chronic lymphocytic leukemia determined by high-pressure liquid chromatography and mass spectrometry. J Am Soc Mass Spectrom.

[B34] Su X, Jacob NK, Amunugama R, Lucas DM, Knapp AR, Ren C, Davis ME, Marcussi G, Parthun MR, Byrd JC, Fishel R, Freitas MA (2007). Liquid chromatography mass spectrometry profiling of histones. J Chromatogr B.

[B35] Perkins DN, Pappin DJC, Creasy DM, Cottrell JS (1999). Probability-based protein identification by searching sequence database using mass spectrometry data. Electrophoresis.

[B36] Xu H, Freitas MA (2008). Monte Carlo simulation based algorithms for analysis of shotgun proteomic data. J Proteome Res.

[B37] Huttlin EL, Hegeman AD, Harms AC, Sussman MR (2007). Prediction of error associated with false-positive rate determinantion for peptide identification in large-scale proteomics experiments using a combined reversed and forward peptide sequence database strategy. J Proteome Res.

[B38] Elias JE, Gygi SP (2007). Target-decoy search strategy for increased confidence in large-scale protein identifications by mass spectrometry. Nature Methods.

[B39] Prince JT, Carlson MW, Wang R, Lu P, Marcotte EM (2004). The need for a public proteomics repository. Nature Biotechnology.

[B40] Fox J (2002). An R and S-PLUS comparison to applied regression.

